# Comparison of artificial neural network analysis with other multimarker methods for detecting genetic association

**DOI:** 10.1186/1471-2156-8-49

**Published:** 2007-07-18

**Authors:** David Curtis

**Affiliations:** 1 Academic Centre for Psychiatry, St Bartholomew's and Royal London School of Medicine and Dentistry, Royal London Hospital, Whitechapel, London E1 1BB, UK

## Abstract

**Background:**

Debate remains as to the optimal method for utilising genotype data obtained from multiple markers in case-control association studies. I and colleagues have previously described a method of association analysis using artificial neural networks (ANNs), whose performance compared favourably to single-marker methods. Here, the perfomance of ANN analysis is compared with other multi-marker methods, comprising different haplotype-based analyses and locus-based analyses.

**Results:**

Of several methods studied and applied to simulated SNP datasets, heterogeneity testing of estimated haplotype frequencies using asymptotic *p *values rather than permutation testing had the lowest power of the methods studied and ANN analysis had the highest power. The difference in power to detect association between these two methods was statistically significant (*p *= 0.001) but other comparisons between methods were not significant. The raw *t *statistic obtained from ANN analysis correlated highly with the empirical statistical significance obtained from permutation testing of the ANN results and with the *p *value obtained from the heterogeneity test.

**Conclusion:**

Although ANN analysis was more powerful than the standard haplotype-based test it is unlikely to be taken up widely. The permutation testing necessary to obtain a valid *p *value makes it slow to perform and it is not underpinned by a theoretical model relating marker genotypes to disease phenotype. Nevertheless, the superior performance of this method does imply that the widely-used haplotype-based methods for detecting association with multiple markers are not optimal and efforts could be made to improve upon them. The fact that the *t *statistic obtained from ANN analysis is highly correlated with the statistical significance does suggest a possibility to use ANN analysis in situations where large numbers of markers have been genotyped, since the *t* value could be used as a proxy for the *p *value in preliminary analyses.

## Background

As discussed recently [1], when genetic markers are used to attempt to detect association with a disease phenotype there are grounds for expecting that, in some circumstances at least, power will be gained by analysing groups of markers jointly rather than considering each marker individually. However uncertainty remains as to the best method for carrying out such a multimarker analysis. Probably the most commonly used approach at present is to carry out haplotype-based analyses, in which haplotypes are estimated from phase-unknown genotypes and then the estimated haplotype frequencies in case and control groups are compared, for example using the GENECOUNTING program [2,3]. From a theoretical point of view this approach may not be optimal for a number of reasons. Typically one will use a likelihood ratio test for heterogeneity of haplotype frequencies and this will have a number of degrees of freedom equal to one less than the number of haplotypes estimated to be present, typically 2^m ^if there are *m *biallelic markers [4]. Using this many degrees of freedom may result in a conservative test. This problem can partly be addressed by permutation-testing to assess statistical significance but it is still clear that the approach is not optimal. For example, suppose there is a 3-marker haplotype associated with disease but one carries out the test including an additional 2 markers which are not associated. Within the 32 possible 5-marker haplotypes there will be 4 which contain the associated 3-marker haplotype but these 4 haplotypes will not be treated as in any way "similar" to each other and the signal from them may well be drowned out by the noise from the other markers. Rather than test for heterogeneity of haplotype frequencies between cases and controls one may seek to model the effects of haplotypes on risk of affection. This different, albeit related, approach is implemented in the UNPHASED program and involves estimating haplotype frequencies and then carrying out logistic regression analysis with the individual haplotypes modelled to confer different risks of affection [5,6].

An alternative approach to utilising multimarker data is to model the effect of each marker separately, generally producing tests with fewer degrees of freedom. A previous investigation [7] compared such locus scoring tests to haplotype scoring tests and found that former were more powerful. In our own investigations [1], we compared a locus-based test implementing logistic regression with a haplotype-based heterogeneity test and found that they had similar power to detect a single pathogenic mutation. The UNPHASED program incorporates an option to treat alleles, rather than haplotypes, as risk factors, resulting in a similar method of analysis [6]. There are theoretical reasons to expect that haplotype-based methods might be relatively more powerful if more than one mutation were present. This is because different haplotypes might be in linkage disequilibrium (LD) with different mutations and distortions in their freqeuencies might be easier to detect than effects on the allele frequencies of individual markers. In this situation one might expect that locus scoring tests would lose power through their failure to consider haplotypic effects.

I and colleagues have proposed an alternative method for analysing multimarker data using artificial neural networks (ANNs) [8,9]. ANNs are designed to detect patterns in input data which may match to output data even if the nature of such patterns is not known *a priori*. By training an ANN to match multimarker genotypes to disease phenotype the hope is that it may be able to detect association which may be based on one marker or several, full haplotypes or partial ones and one or more different haplotypes. We showed that this method can be more powerful than tests based on single markers but it has not previously been compared it with other multimarker approaches.

Here, I investigate the relative power of haplotype analysis, logistic regression, UNPHASED analyses and ANN analysis using real genotype data to provide information on SNP allele frequencies and LD relationships such as would be found in case-control association studies.

## Results

The main results obtained from this investigation are displayed in Table 1. A number of observations are worthy of note.

**Table 1:** Relative power of heterogeneity tests, logistic regression, UNPHASED analyses and ANN analysis to detect association at p < 0.01 in a case control study using different disease models

Chromosome	Penetrance values	Power of heterogeneity test of haplotype frequencies	Power of heterogeneity test using permutation testing	Power of logistic regression analysis	Power of UNPHASED analysis using haplotype effects	Power of UNPHASED analysis using allele effects	Power of ANN analysis
7	0.01, 0.02, 0.02	0.48	0.50	0.50	0.54	0.48	0.52
7	0.01, 0.03, 0.03	0.58	0.60	0.61	0.60	0.59	0.68
17	0.01, 0.02, 0.02	0.29	0.30	0.31	0.31	0.29	0.34
17	0.01, 0.03, 0.03	0.36	0.37	0.36	0.37	0.36	0.44
7	0.01, 0.01, 0.02	0.37	0.43	0.38	0.42	0.40	0.46
7	0.01, 0.01, 0.03	0.48	0.48	0.48	0.52	0.51	0.60
17	0.01, 0.01, 0.02	0.31	0.33	0.34	0.37	0.35	0.34
17	0.01, 0.01, 0.03	0.54	0.54	0.57	0.58	0.58	0.58
All combined		0.42	0.44	0.44	0.45	0.44	0.48

Firstly, in terms of absolute power we can see that for samples consisting of a few hundred cases and controls association may well not be detected even using closely spaced SNPs. Of course, the ability to detect association is crucially dependent on sample size and disease model as well as LD relationships between polymorphisms. The disease models used here incorporate relative risks of 2 or 3 and with these sample sizes power to detect association at *p *< 0.01 ranges from 29% to 68%.

For the haplotype-based test, with the exception of a couple of datasets in which power was equal, permutation testing was always more powerful than referring to the asymptotic chi-squared distribution, in most cases to only a small degree although in one case with a power difference as high as 6%. By this we mean that the permutation test more often produced a *p *value of 0.01 or less. The minimum empirical *p *value that can be estimated with 999 permutations is 0.001 and the asymptotic test often produced a much lower value than this, meaning that the average *p *value for the asymptotic test was more highly significant. Nevertheless, in terms of ability to reach the threshold set *a priori *permutation testing was more powerful. This indicates that using the asymptotic distribution does produce a test which is somewhat conservative.

The power was very similar for the permutation-based test of heterogeneity of haplotype frequencies, the logistic regression test for effects of individual loci and both the haplotype-based and allele-based analyses implemented in UNPHASED. In fact, the power of the haplotype-based analysis used by UNPHASED was consistently higher than that of the allele-based analysis but only to a small extent, amounting on average to a difference of only 1%.

The ANN analysis tended to have a higher power than other methods, albeit not consistently so in that for a couple of models the haplotype-based analysis implemented in UNPHASED had higher power. Across all models, the difference in power between ANN analysis and the heterogeneity test of haplotype frequencies based on asymptotic *p *values was statistically significant (chi-squared = 10.4, 1 df, *p *= 0.0012). However, other comparisons between tests were not statistically significant. The power advantage of the ANN analysis over the heterogeneity test of haplotype frequencies was higher for the models in which the relative risk was set to 3 rather than 2 (*p *= 0.03) and for those using the more closely spaced markers (*p *= 0.01) but there was no overall difference in power between dominant and recessive models.

There was a fairly strong correlation between the *p *values obtained from the ANN analysis and those obtained from the heterogeneity test of haplotype frequencies (R = 0.71). There was also a high correlation between the *t *statistic obtained from ANN analysis and the empirical *p *value (R = 0.91), suggesting that the *t *statistic could be utilised as a preliminary indicator for genetic association without the necessity to carry out permutation testing. In support of this notion, this *t *statistic also demonstrated high correlation (R = 0.86) with the *p *values obtained from the heterogeneity test of haplotype frequencies.

## Discussion

We must reiterate that the results we have obtained are contingent on utilising particular disease models and sample sizes, though the SNP data we have used do reflect real data in terms of marker informativeness, spacing and LD relationships. That said, there do seem to be some interesting implications. Haplotype-based analysis is currently the most widely used method and can probably be fairly regarded as standard. However under the conditions of this investigation it is shown to be by no means the most powerful method and is out-performed by logistic regression, UNPHASED and ANN analysis. When the asymptotic *p *value is used for the haplotype analysis, as in practice would usually be the case for an initial screen, ANN analysis has a power advantage which would have practical implications in the real world. When one considers the vast resources which can go into performing a case-control association study it would be disastrous for an association to be missed through utilising a test which was several percentage points less powerful than another.

Quantifying relative power by assessing the proportion of times each method of analysis yields a particular target *p *value might imply that one was taking a very simplistic view of how genetic investigations were carried out. This would be that a set of markers were genotyped and then analysed using only one method of analysis in groups containing a number of markers which had been specified in advance. Regions containing a group which reached the target *p *value would then be subjected to intensive study in an effort to identify variants directly influencing risk of affection while other regions would be ignored. In practice the situation would likely be far more complicated. A variety of methods of analysis might be used, including single marker and multi-marker analyses containing different numbers and combinations of markers, probably selected on an *ad hoc *basis. If a *p *value just failed to reach some arbitrary level of significance then it would not simply be ignored but the region might be kept under consideration for the future, although afforded a lower priority. Regions yielding the most highly significant results would probably be examined first but the failure to reach a target *p *value would not necessarily mean the difference between detecting an association and missing it entirely.

With this *caveat *in mind, it does nevertheless seem that this investigation demonstrates that haplotype based analysis, as commonly used, is at least in some situations not the best way to detect association using multi-marker data. This would confirm what might expect from theoretical considerations, in particular that there is a failure to treat haplotypes which are similar to each other any differently from those which have no alleles at all in common. The pattern-matching abilities of ANN analysis do seem better able to detect the kinds of deviation from random distribution of multi-marker unphased genotypes which are generated when a susceptibility locus is present and in LD with at least some of the marker loci. There is a suggestion from these results that the advantage of ANN analysis may be more pronounced when there is a larger genetic effect and when markers are closer together. However further work would need to be carried out to formally investigate whether there were particular situations in which one the different methods would have different relative merits. As we have mentioned previously [1], a particular situation worthy of study would be that in which different mutations in the same gene can influence risk. From a theoretical point of view one might well expect this to have an important impact but modelling this situation would require more sophisticated simulation software.

Although ANN analysis demonstrates superiority in this investigation, it would be unrealistic to recommend that it be widely adopted. One disadvantage is the lack of theory-driven testing for association. The ANN detects some kind of patterns which can be used to distinguish the genotypes of cases from those of controls but the nature of this association is not specified in advance and even after the ANN has been trained to detect a difference the criteria it uses are unclear. This is not a desirable situation. The ANN can output a lists of the genotypes which produce the highest and lowest outputs and perusing these lists may offer some indication of which alleles and combinations of alleles appear to be commoner in cases but this is hardly a rigorous process. There is also a very important practical disadvantage, which is that ANN analysis is slow. For each set of markers one has to go through a cycle of repeated trainings followed by testing and then these cycles need to be repeated many times on permuted data to obtain an empirical *p *value. As currently implemented, the ANN takes in the region of 20–30 minutes to analyse one set of markers in a few hundred subjects using an ordinary desktop PC and 999 permutations. If one wished to estimate a lower *p *value, in the region of 0.001, one would need 10 times as many permutations and the analysis would take 10 times as long. One approach which can produce some useful speed benefits is to use sequential sampling to obtain empirical *p *values [10]. When carrying out permutation testing, rather than setting the number of permuted replicates, *n*, to a fixed number one instead sets a target for *r*, the number of times that a permuted replicate should exceed the test statistic obtained from the real dataset. Typically a target for *r *might be set to a value of 10 or 20. One would also set some maximum value of *n *to ensure that the procedure did eventually finish. If the target value for *r *is reached then the empirical significance is given by *p *= *r*/*n *while if the target is not reached before *n *reaches its maximum value the empirical significance is given by *p *= (*r*+1)/*(n*+1), as used in conventional Monte Carlo testing. This produces a very valuable increase in speed of permutation testing when the *p *value to be estimated turns out to be non-significant. If there is no association present then one will expect to only perform 2*r *permutations before the target is reached. With a target of *r *= 10 then one achieves a 50-fold speed increase compared with using the conventional method with *n *= 999. This approach would be very useful when analysing large numbers of markers, most of which are expected not to demonstrate association. However sequential sampling does not provide any advantage when the *p *value to be estimated is in fact small and one will still need to carry out a large number of permutations in order to produce an acceptably accurate estimate. In some genetic investigations multi-marker analysis produces *p *values which are very small indeed and it would be difficult to obtain these using a Monte Carlo approach. If one carries out a screen using hundreds of thousands of markers then one will not wish to set a threshold of *p *≤ 0.01 to designate regions for further consideration, since this would leave one with thousands of candidate regions. However such a threshold might arguably be appropriate if a small number of markers were investigated in a region which was already of interest *a priori *and it could be noted that in the current investigation *p *values of this magnitude were sometimes produced from a sample of several hundred subjects and with a disease locus having a moderate effect on risk.

An alternative approach to implementing ANN analysis for large numbers of markers might be to consider the raw *t *statistic as an indicator of association rather than going on to carry out permutation testing. We have previously emphasised that, because the *t *statistic is obtained by testing the same case and control samples as were used to train the ANN, no formal interpretation can be made for the magnitude of evidence in favour of association from the *t *statistic on its own. It is simply a measure of how well the network has been able to adapt to the data it has been presented with in terms of finding an algorithm which will match inputs with outputs. A number of confounding factors might theoretically be expected to influence this ability, in particular the allele frequencies of the markers used and the LD relationships between them. However, here we have shown that such concerns may in fact be exaggerated and that the *t *statistic is in itself a reasonable indicator of association. It is highly correlated both with the empirical *p *values obtained from permutation testing of ANN analysis (R = 0.91) and with the *p *obtained from conventional tests for heterogeneity of haplotype frequencies (R = 0.86). In practice this means that if one were to study thousands of markers one might begin by carrying out ANN analysis without permutation testing, knowing that those sets of markers producing the highest values for the *t *statistic were likely to be those showing the strongest evidence for association. One could then select these sets and carry out permutation testing on them in order to obtain a formal measure of statistical significance.

## Conclusion

This investigation demonstrates that in at least some situations standard haplotype-based analysis is less powerful than other methods. Although ANN analysis has performed better we do not envisage that it will be widely taken up as an alternative. However, the results do suggest that there is room to develop new methods which might share the advantages of ANN analysis in terms of implementing a parsimonious approach to detect the patterns of multi-marker genotypes which can be observed when an associated susceptibility locus is present. Such methods might offer useful increases of power. Given the resources which need to be invested in collecting samples of cases and controls and obtaining genotypes it seems sensible to argue that considerable effort should be expended on ensuring that methods of analysis applied to the data obtained are as effective as possible.

For now, we suggest that it should be recognised that heterogeneity tests of haplotype frequencies may be intrinsically somewhat conservative. This would imply that when analysing many markers one might set a somewhat lower threshold to indicate which markers were worthy of further investigation, which would include formal permutation testing in order to obtain a reliable *p *value. With the exception of ANN analysis, which may have a slight advantage, the other methods studied demonstrate similar perfomance to each other under the condtions of these simulations. Investigators could feel reassured about using any of them until further information regarding their relative performance becomes available.

## Methods

We have described the general approach in more detail previously [1]. In essence, it consists of using real SNP genotypes from the HAPMAP project [11] to produce simulated data for a case-control study based on observed SNP allele frequencies and LD relationships. The simulated genotypes are then analysed by different methods and the results compared. The scenario envisaged is that one SNP affects susceptibility to disease but has not been genotyped. Available to the investigator are the phase-unknown genotypes of 4 nearby markers and the aim is to detect association with the disease phenotype in the context of a case-control association study.

### Original SNP genotypes

The two sets of markers were downloaded from the HAPMAP site [12] from regions of chromosomes 17 and 7. Genotypes were available from 60 unrelated subjects, who are parents in the 30 trios comprising the CEPH dataset. SNPs were used from a non-ENCODE region of chromosome 17 spanning approximately 240 kb over 40247240–40493936. They were chosen to have minor allele frequency >5%, yielding 62 SNPs with an average distance between them of 4 kb. The chromosome 7 SNPs were located in a region of chromosome 7 spanning 109 kb over 2693665–2462902 which had been studied by the ENCODE Consortium [13] which had made intensive efforts to identify all available SNPs, meaning that SNPs in this region would be more closely spaced. Again, SNPs were selected to have minor allele frequency >5%, yielding 64 SNPs with an average spacing of just 1.7 kb. Any SNP which was in complete LD with any other was then discarded, leaving 40 chromosome 17 and 30 chromosome 7 SNPs.

### Estimation of disease-marker haplotype frequencies

For each of the two regions we selected each SNP locus in turn to act as a disease susceptibility locus. For each selected disease locus we then used 4 adjacent loci to act as markers, using a sliding window ranging from the 4 SNPs on one side of the disease locus to the 4 on the other. This meant that, except for SNPs at the ends of the dataset, 5 sets of 4 markers were used for each disease locus. For each set of disease and marker loci I then estimated the haplotype frequencies in the observed HAPMAP genotypes using the SNPHAP program [14], which provides maximum likelihood haplotype frequencies from unphased multilocus genotypes. These real haplotype frequencies were then used to generate simulated datasets such as might be observed in case-control studies were the marker(s) to be typed in a sample in which the disease locus exerted an effect on susceptibility.

### Simulation of genotypes

For each disease locus 4 different transmission models were used by considering a dominant or recessive effect and a relative risk of 2 or 3. A penetrance of 0.01 was used for subjects having no copies of the disease allele, while for subjects having one (dominant) or two (recessive) copies of the disease allele the penetrance was set to 0.02 or 0.03. The allele frequencies of the disease locus were taken to be the observed frequencies of the SNP under consideration. As described previously [1] the expected proportions of cases and of controls having 0, 1 or 2 copies of disease allele were calculated using this transmission model. A simulated sample of cases and controls was then generated. Each case or control was first allocated a number of disease alleles according to probabilities equal to these expected proportions. Then two haplotypes bearing this number of disease alleles were sampled at random according to the estimated haplotype frequencies obtained from the SNPHAP program. The number of cases and controls generated was pragmatically chosen to yield power to detect association ranging between 25% and 60%. In the event sample sizes were either 300 or 400 each of cases and of controls, depending on whether the penetrance was set to 0.03 or 0.02.

### Analysis of simulated genotypes

The 4-marker phase-unknown genotypes obtained from the above procedure were analysed using a heterogeneity test of haplotype frequencies, logistic regression, UNPHASED analyses and ANN association testing. For each test the ability to detect assocation at *p *< 0.01 was recorded. For heterogeneity testing the GENECOUNTING program and RUNGC support program were used [3,4]. The GENECOUNTING program estimates maximum likelihood haplotype frequencies from unphased multilocus genotypes, which may include multiallele genotypes and missing data. It also outputs a log likelihood for the dataset assuming these frequencies. The RUNGC program constructs a test for heterogeneity of haplotype frequencies by obtaining these maximised log likelihoods for the controls, the cases and the combined dataset. A likelihood ratio statistic (LRS) is derived as 2(L_CASE_+L_CONTROL_-L_COMBINED_) and this is taken as a chi-squared statistic with degrees of freedom equal to the difference in the number of haplotypes estimated to have non-zero frequency in the cases plus the controls compared to the combined dataset. The RUNGC program can also carry out permutation testing to obtain an empirical significance for this LRS and for the present investigation I used 999 permutations. The proportion of times the LRSfrom the real data is exceeded by that obtained from permuted data provides an empirical *p *value using the formula *p *= (*r*+1)/(*n*+1) where *r *is the number of times the real statistic is exceeded and *n *is the number of replicates, here 999 [15,16]. Using 999 replicates means that a *p *value of 0.01 can be estimated with reasonable accuracy [10,15]. In order to speed up the process by reducing the number of permutations required for some datasets which clearly did not provide evidence for association, sequential Monte-Carlo testing was implemented [10]. This meant that a target number of *r *= 10 was set for the real statistic to be exceeded. If this was met before all 999 replicates had been performed then the number of replicates taken to achieve this was recorded as *n *and the empirical *p *value was calculated as *r*/*n*. As in our previous investigation [1], logistic regression analysis was used to test for the main effects of each marker locus. The A allele at each SNP was arbitrarily chosen as a risk factor which might influence risk so that genotypes AA, AB and BB would correspond to exposure of 2, 1 or 0. No interaction terms of independent variables were included in this analysis. This method had been implemented within the simulation program and logistic regression was carried out to estimate the log likelihoods for the dataset assuming no genetic effect on risk or assuming that the risk allele at each locus exerted an independent effect, producing a LRS having 4 degrees of freedom.

Two additional methods of analysis were carried out using the UNPHASED program [5,6]. The first implemented the "full" model, in which each possible haplotype is considered independently. The population haplotype frequencies are estimated from the controls. Each subject is then assigned a number of possible haplotypes which they may possess with different probabilities. Logistic regression is carried out with the haplotypes taken to be risk factors and each subject's exposure to the risk factor is weighted according to the probability of possessing that haplotype. Log likelihoods are obtained under the assumption that the haplotype has no effect on risk and under the assumption that each haplotype has a separate effect on risk, again yielding an LRS. The second method carried out using the UNPHASED program consisted of a logistic regression analysis similar to the one described above. Log likelihoods were used to compare the hypothesis that there is no genetic effect on risk and the hypothesis that alleles at each locus act in a multiplicative function to influence risk.

For the ANN analysis previously described software was used (available from our website [17]) and a network was constructed having 4 input nodes (one for each marker genotype, coded 0, 1 or 2), two hidden layers each containing three nodes and one output node. Each node was connected to every node in the next layer. The output of each node was obtained by applying a logistic activation function to the sum of the weighted inputs to that node. The network was trained over 100 cycles using the marker genotypes as inputs and the affection status, coded as 0 for controls and 1 for cases, as target output. A standard back propagation training algorithm was used and 200 training sets were run [8]. During these, the weights of connections between nodes of the network would be adjusted along with thresholds for the activation function of each node, the aim being to have the output associated with each set of inputs match as closely as possible to the target output. Once the network had been trained a test run was performed in which the genotypes were again input and the outputs for cases and controls were used to produce a conventional *t *statistic. This provides a measure of the extent to which the network has been able to "learn" to distinguish cases from controls based on marker genotypes. Because the training and testing are carried out on the same dataset it was not expected that the statistical significance for evidence in favour of association could be obtained by referring this *t *statisticto the asymptotic distribution. Rather, the genotype and case-control status were randomly permuted and the whole process was repeated for 999 replicates, each yielding its own *t *statistic. This allowed one to assess the extent to which the ability to distinguish cases from controls based on marker genotypes differed in the real dataset from that which might be expected by chance. Again, the statistical significance of the test for association was given by the proportion of times the *t *statistic from the observed data was exceeded by one from a permuted replicate [9], unless this occurred on *r *= 10 occasions using only *n *replicates (*n *< 999) in which case the empirical *p *value was calculated as *r*/*n *[10].

### Comparisons between methods of analysis

For each disease model, 130 datasets were analysed using chromosome 7 SNPs and 180 using the more widely spaced chromosome 17 SNPs. Thus, in all 1240 simulated datasets were analysed. This was a time-consuming process because ANN analysis is intrinsically slow as it involving training the network repeatedly on many permuted datasets. Results were considered for each model and pooled across models. The correlations of the *p *value produced from ANN analysis with haplotype analysis and with logistic regression analysis were calculated, as were correlations between the raw *t *statistic from the ANN analysis with the empirical *p *value obtained from permutation of ANN results and the asymptotic *p *value for the haplotype analysis. For each method the proportion of times the analysis achieved a *p *value of 0.01 was recorded. Formal tests of differences of power between pairs of methods were compared using a 2-by-2 chi-squared test. For correlation analyses -log10(*p*) was used except that for *p *values obtained by permutation testing ranks were used because -log10(*p*) would have a ceiling of 3.

## Abbreviations

ANN – artificial neural network

LD – linkage disequilibrium

SNP – single nucleotide polymorphism

## Authors' contributions

DC carried out the simulations and analyses and wrote the paper.
